# Inflammatory Process Modulation by Homeopathic *Arnica montana* 6CH: The Role of Individual Variation

**DOI:** 10.1155/2011/917541

**Published:** 2011-01-19

**Authors:** Ana Paula Kawakami, Cesar Sato, Thayna Neves Cardoso, Leoni Villano Bonamin

**Affiliations:** ^1^Laboratory of Cell and Molecular Biology, Research Center of University Paulista, Avenue José Maria Whitaker, 290, 04057-000 São Paulo, SP, Brazil; ^2^Laboratory of Veterinary Pathology, University of Santo Amaro, 04829-300 São Paulo, SP, Brazil

## Abstract

The effects of *Arnica montana* 6cH on the individual modulation of acute inflammation kinetics in rats were evaluated. Adult male Wistar rats were inoculated with 1% carrageenan into the footpad and treated with *Arnica montana* 6cH, dexamethasone (4.0 mg/kg; positive control) or 5% hydroalcoholic solution (negative control), *per os*, each 15 minutes, between 30 and 180 minutes after the irritant inoculation. Histopathological and immunohistochemistry procedures were done in order to get a panel of inflammatory positive cells for CD3 (T lymphocytes), CD45RA (B lymphocytes), CD18 (beta 2 integrin), CD163 (ED2 protein), CD54 (ICAM-1), and MAC 387 (monocytes and macrophages). The statistical treatment of data included a *posteriori* classification of animals from each group (*N* = 20) in two subgroups presenting spontaneous *precocious* or *late* oedema. Animals that presented precocious oedema were less responsible to *Arnica montana* 6cH in relation to hemodynamic changes. Instead, rats that exhibited *late* oedema presented less intense oedema (*P* = .01), lower percentage of mast cell degranulation (*P* = .0001), and increase in lymphatic vessels diameter (*P* = .05). The data suggest an individually qualitative adjustment of inflammatory vascular events by *Arnica montana* 6cH.

## 1. Introduction

One of the most discussed controversies about the efficacy of homeopathic medicines is the necessity to identify a perfect symptomatic analogy between patient and drug pathogenesy, that is, the necessity of individual prescription according to the similia principle. This particular feature is one of the most difficult challenges of the scientific research in this field because of the technical difficulty of its experimental demonstration. Indeed, few experimental studies about are found in the literature [1–6]. Thus, the experimental demonstration of this particularity and the comprehension of its mechanisms can be useful tools to solve the chronic controversies about homeopathy efficacy, often seen in clinical trials [7, 8]. Recently, an elegant *in vitro* study demonstrated the importance of the regulatory and adaptive cell mechanisms in the magnitude of homeopathic remedies inducing cytotoxic effects on cancer cells [9].

From 1997 up to 2008, we have developed a step-by-step research trial about the biological effects of homeopathic *Arnica montana*, specially the *Arnica montana* 6cH, using animal models [10–13]. In short, the results obtained in these studies show that *Arnica montana* 6cH is able to modulate the acute inflammatory process in rats, since it can increase lymphatic oedema absorption and local blood flow, as well as to promote the array of polymorphonuclear cell migration. All these experiments are chronologically described in [13].

The *Arnica montana* is a plant belonging to the Compositae family that grows on the hills of East and Central Europe. Several active compounds are identified in its leaves, flowers, and roots, such as alcohols, tannin, flavonoids, and sesquirterpenic lactones, especially helenalin [14]. The main action of helenalin is the inhibition of the transcription factor NF*κβ*, similarly to corticoid steroids [15]. The incidental ingestion of the plant can cause vasodilatation, blood stasis, hemorrhage, oedema, and pain. These effects are the main topics described in the *Arnica montana materia medica* [14]. Because of that, trauma pain and oedema absorption are the main indications for the clinical and experimental use of homeopathic preparations of *Arnica montana* [16–20]. Recently, other uses of high diluted *Arnica montana* have been proposed, like the agronomic use of Arnica in the potencies 3, 6, and 12cH to improve plants growth [21].

The aim of the present study was to check two hypotheses: (a) the putative modulation of vascular and cell events in acute inflammation by Arnica, focusing on the rate of neutrophils, monocytes, T and B lymphocytes present in the inflammatory site, and the intensity of adhesion molecules expression in inflamed connective tissue, using immunohistochemical and histomorphometrical techniques; (b) the putative interference on the outputs of the natural individual variations of inflammation kinetics.

## 2. Material and Methods

### 2.1. Animals

Male adult Wistar rats, weighting between 250 and 300 g, were used. Rats were maintained in conventional laboratory polypropylene cages (5 to 7 animals per cage), with controlled temperature (25 ± 3°C) and light cycle (lights on from 06:00 AM to 06:00 PM). Water and food were offered *ad libitum*. Prior to the beginning of the experiment, animals were randomly weighed, separated, and identified in three groups of 20 rats each.

### 2.2. Induction of Acute Inflammation

The acute inflammatory process was induced by the subcutaneous inoculation of 1% *kappa* carrageenan (SIGMA) diluted in sterile saline into the footpad, whose thickness was previously measured with a micrometer (MYTUTOYO).

After 30 and 180 minutes, new measurements were made, in order to evaluate the pre- and posttreatment oedema evolution in function of time. Thus, the first 30 minutes measured the spontaneous oedema formation and the remaining 150 minutes measured the drug effects, since treatments were done after the 30-minute measurement. At 180 minutes, animals were euthanized by cervical traction under deep anesthesia, and foot pads were harvested and fixed in buffered 10% formaldehyde during 24 hours—maximum—before being processed by conventional histological techniques, including paraffin embedding, hematoxylin-eosin, and toluidine blue staining. The same paraffin blocks were used to perform the immunohistochemical procedures. For each footpad, one single slide was done.

### 2.3. Groups and Treatments

Each group of rats was treated with a specific substance, according to Table 1. Rats treated with *Arnica montana* 6cH (experimental) and unsuccussed 5% hydroalcoholic solution (negative control) obtained from the same supplier were identified by codes, in a manner that all treatments and measurements were made in blind. These codes were created by a laboratory technician who did not participate of the study and kept in a sealed sheet up to the statistical analysis, when they were revealed.

The dexamethasone-treated rats (positive control) were made openly, because of the physical characteristics of the used formulation; it was quite different from the homeopathic ones and, thus, easily recognizable. The treatment and the microscopic analysis of samples were performed by two independent persons, always in blind. The total dose of dexamethasone used to produce anti-inflammatory effects was 4 mg/kg, according to the standards defined previously [22]. This fractioned protocol was designed to reproduce a usual homeopathic clinical condition of acute inflammation treatment and was also used in the previous studies performed by our group [13]. Dexamethasone was chosen as a positive control group because its mechanism of action mimics the mechanism of helenalin, the main active principle of *Arnica montana* [13, 15].

The commercial *Arnica montana* 6cH was prepared in 5% hydroalcoholic solution by a pharmacy certified by the regulatory Brazilian National Agency for Sanitary Vigilance (ANVISA), *Farmácia Sensitiva*, São Paulo. The techniques used in the preparation were according to the *Brazilian Homeopathic Pharmacopea*, 2nd Edition, 1997.

### 2.4. Data Analysis

After the first plotting of the oedema intensity obtained during the pretreatment period (from zero to 30 minutes), data were classified in crescent ordering using an Excel 2003 software, in a way that it would be possible to divide, for each group, the 10 rats (50%) that presented spontaneously lower oedema intensity and the 10 rats (50%) that presented higher oedema intensity. Thus, two subgroups of 10 animals were formed for each experimental group, characterizing two different rat subpopulations according to inflammatory kinetic pattern (Table 2). The whole experimental design is explained in a flowchart (Figure 7).

Since the classic curve of carrageenan-induced oedema is over up to 6 hours after inoculation, the choice of both times (30 and 180 minutes) was based on its known *plateau *[23, 24]. Moreover, previous studies about the effects of Arnica 6cH upon carrageenan-induced oedema show no more effects after this period [10, 11]. The kinetics of inflammatory process evaluation after the injection of carrageenan are a classical experimental model developed during the 70 s [23, 24]. Thus, it is a trustful criterion to be used in this case.

**Figure 1:**
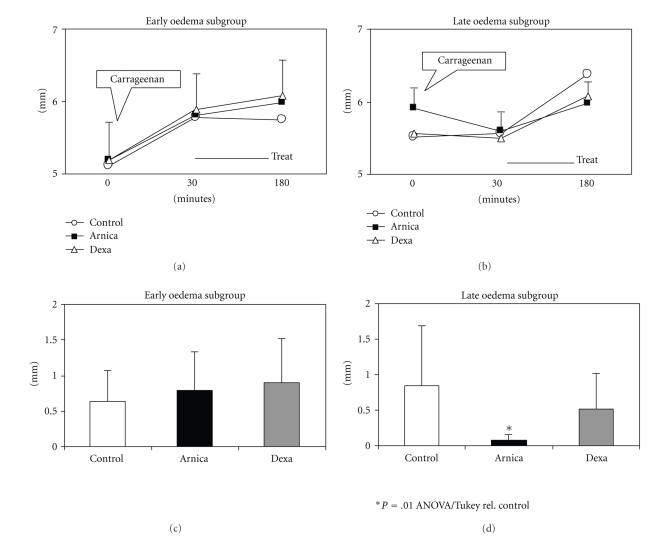
Oedema (mm) in different groups and subgroups. (a) Evolution of oedema in function of time in the *early oedema subgroup*; (b) evolution of oedema in function of time in the *late oedema subgroup*; (c) intensity of oedema at the end of the experiment in the *early oedema subgroup*; (d) intensity of oedema at the end of the experiment in the *late oedema subgroup*. *P = .01; ANOVA, Tuckey-Krammer, in relation to control. The values represent mean ± standard deviation.

After plotting data (Figures 1(a) and 1(b)), both kinetic patterns became easy to identify: (a) animals that presented less intense oedema during the first pretreatment 30 minutes had the peak between 30 and 180 minutes (named *late oedema*) and (b) those that presented more intense oedema in the first 30 minutes decreased after this period (named *early oedema*).

All results were compared among the six subgroups. This *a posteriori* selection was made in order to check the role of individual idiosyncrasy in the Arnica effect according to the similia principle, since the speed of oedema remission is one of the most known *Arnica montana* symptoms described in *materia medica* [10, 11, 13, 14].

### 2.5. Immunohistochemical Analysis

All slides were washed with 1 : 1 alcohol-ether solution during five minutes and then dried with a soft sheet of paper and treated with 1 : 10 poly-L-lysin (SIGMA). Then, 5 microns paraffin-embedded tissue slices were transferred to their surface using a 40°C histological bath. Next, slices were deparaffined through two baths in absolute xylol for 2 minutes and two baths in absolute alcohol for 3 minutes. Slides were, then, washed in current tap water.

For the immunohistochemistry procedures, a first step for antigen retrieving was made using heat treatment of samples. Slides were put inside a citrate buffer bath (SIGMA), pH = 6.0, containing 0.5% tween 20 (DAKO), and heated in an electric pot at 80°C (PANASONIC) during 20 minutes. After that, slides were washed in PBS, pH = 7.2 (SIGMA), for 6 minutes and dried with a soft absorbent paper, and the tissue sample was delimited using an appropriate pen (Pap-pen, AbCam).

The endogenous peroxidase activity was blocked by incubation of tissues in a 3% H_2_O_2_ solution diluted in methanol (ISOFAR), for 15 minutes at room temperature. Then, they were washed in PBS (SIGMA) for 6 minutes and treated with 2.5% horse normal serum (VECTOR) during 20 minutes at 25°C, for blocking unspecific protein-binding sites. Immediately after this step, the tissues were treated with the primary antibody (see dilutions and specifications in Table 3) and left standing overnight at 4°C in a humid chamber. All dilutions of primary antibodies were made in 1% bovine serum albumin (BSA).

In the next day, cuts were washed in PBS (sigma) for 6 minutes and treated with the polymer-peroxidase-conjugated secondary antibody (IMPRESS UNIVERSAL, VECTOR), at 25°C during 30 minutes. After a new washing in PBS (6 minutes), they were exposed to DAB (DAKO) for 3 seconds, washed once again in tap water, stained with Harris hematoxylin (01 minute), and mounted.

### 2.6. Histomorphometry

For the evaluation of mast cell degranulation percentage, two hundred cells per slide stained by Toluidine blue method were counted, using contiguous fields, differentiating the degranulated from the nondegranulated ones. To evaluate the lymphatic vessels diameter, five fields chosen by chance were evaluated using a 200 x objective. Photomicrographs of each field were made (CANNON), and the percentage of lymphatic vessels area per field was determined by a digital image analysis system (Image Tool 3.0). The diameter of lymphatic vessels can be considered as a good parameter to evaluate lymphatic oedema absorption, according to previous observations [13]. As the protocol was about acute events, the hypothesis of lymphangiogenesis was not considered.

For the anti-CD45RA, CD3, CD18, CD163, and MAC 387 markers, ten microscopic fields chosen by chance were observed per slide, using immersion objective (magnitude 1000 x). They included almost all the subcutaneous vascular connective tissues of the pad, and the number of positive cells per field was recorded. In the case of Anti-MAC 387, only mononuclear positive cells were considered. The hematoxylin-eosin-stained slides were used to count the number of PMN cells per field. In this case, the recognition of cells was made only by morphological criteria. The expression of CD163 is proportional to the macrophage maturation, because of that, these results were expressed as the ratio between CD163/MAC 387 mononuclear positive cells per field.

For the Anti-CD54, the intensity of positivity on the endothelial cells surface was evaluated by a score system, varying from 1 to 4. Five fields chosen by chance were evaluated by slide, using immersion objective (magnitude 1000 x), and the scores were done by two independent observers, in a blind manner, to avoid subjective interpretation in the analysis. The final score for each slide was equal to the sum of the partial scores. The criteria of score grading are represented in Table 4.

### 2.7. Statistical Analysis

The Bartlett test was firstly employed to determine the Gaussian distribution of the majority of data points. Then, ANOVA/Tuckey-Krammer or Kruskall-Wallis/Dunn was performed, according to the Bartlett results. The evaluation of mast cell degranulation, instead, was evaluated by *X* ^2^ test. The values of *P* ≤ .05 were considered significant. All statistical analysis was performed using the INSTAT 3 software.

In relation to oedema, an additional intragroup statistical analysis was also performed, and the comparison of oedema intensity among subgroups at 30 minutes (spontaneous oedema) revealed significant differences (Tuckey-Krammer, *P* ≤ .01), validating the proposed experimental design.

### 2.8. Bioethical Criteria

The protocol was approved by the Bioethics Committee of *Universidade Paulista* (protocol 011/07), according to the São Paulo State law no. 11.977/05 (Animal protection code of São Paulo State, Brazil). This procedure is in accordance with the European Convention for the Protection of Vertebrate Animals used for Experimental and Other Scientific Purposes and its appendix.

## 3. Results

The histopathological aspect of inflamed footpads revealed the classic framework of a typical early acute inflammation: oedema and cell infiltrate corresponding to 2/3 of PMN cells and 1/3 mononuclear cells. However, considering the intragroup statistical treatment, the existence of different patterns of inflammatory process development in function of time could be identified (Tables 5 and 6). Thus, there was the necessity to systematize and classify these patterns before analyzing the effects of *Arnica montana* 6cH.

**Figure 2:**
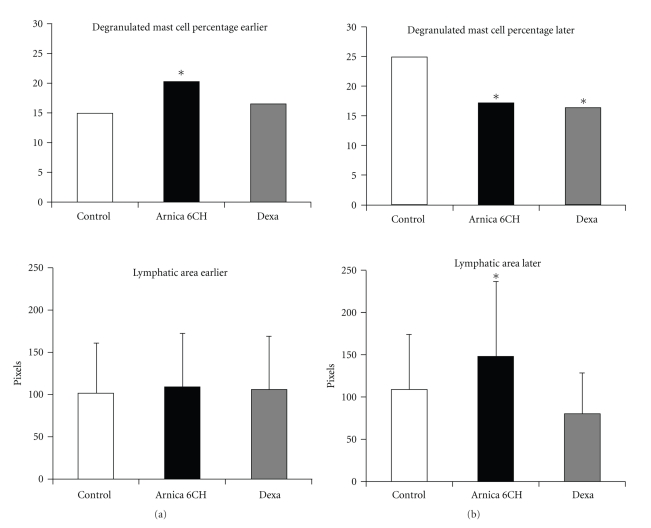
Histogram representing the percentage of degranulated mast cells and lymphatic area (pixels) per field. (a) Subgroup that developed earlier oedema; (b) subgroup that developed later oedema. *X ^2^, *P* = .0001 in relation to control. **Kruskal-Wallis, *P* ≤ .05 in relation to dexamethasone group.

According to that, a significant antioedematous effect of *Arnica montana* 6cH could be seen (ANOVA, *P* = .01) only in the subgroup of rats that presented the peak of oedema between 30 and 180 minutes after the injection of carrageenan into the footpad (called *late subgroup*) (Figure 1). Also, the histomorphometric analysis of slides stained by Toluidine blue revealed that the *late subgroup* also presented less intense mast cell degranulation (*X* ^2^, *P* = .0001) and greater diameter average of lymphatic vessels (Kruskal-Wallis, *P* ≤ .05). Instead, in the *early subgroup* (oedema peak before 30 minutes), only a discrete but significant increase in mast cell degranulation was observed (*X* ^2^, *P* = .0001), as well as increase in scores of CD54 expression by endothelial cells (Kruskal-Wallis, *P* = .05) (Tables 5 and 6, Figures 2 and 5).

**Figure 3:**
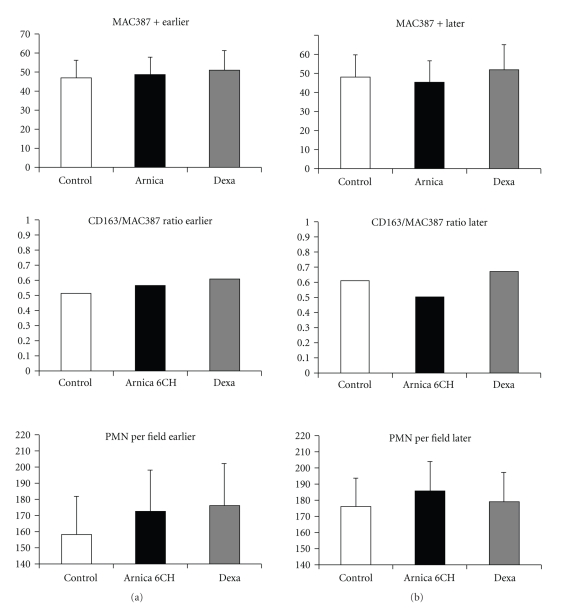
Histogram representing the number of MAC387 positive cells per field, the CD163/MAC387 ratio and the number of polymorphonuclear cells per field. (a) Subgroup that developed earlier oedema; (b) subgroup that developed later oedema. ANOVA, without significance.

**Figure 4:**
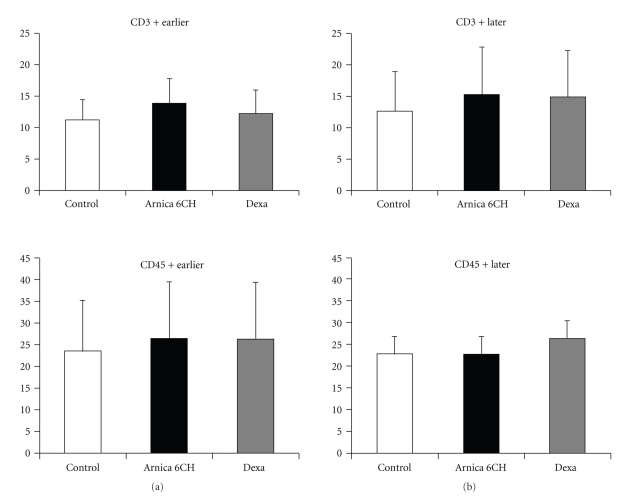
Histogram representing the number of CD3 and CD45R positive cells per field. (a) Subgroup that developed earlier oedema; (b) subgroup that developed later oedema. ANOVA, without significance.

**Figure 5:**
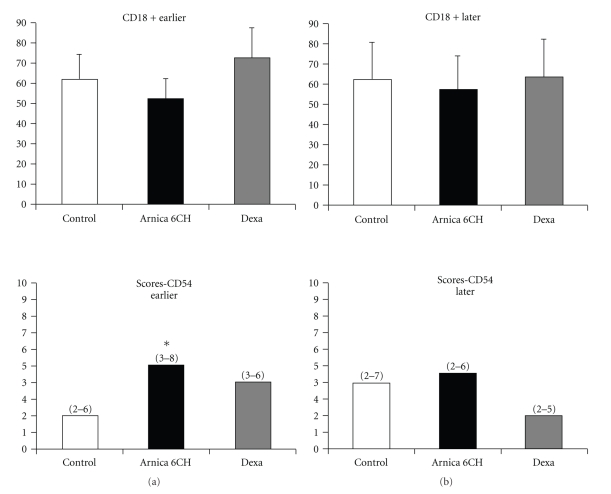
Histogram representing the number of CD18 (beta 2 integrin) positive cells per field and the median (interval) of scores of CD54 (ICAM) labeling. (a) Subgroup that developed earlier oedema; (b) subgroup that developed later oedema. ANOVA, without significance; *Kruskal-Wallis/Dunn, *P* = .03 in relation to control.

No difference among groups and subgroups was observed regarding cell migration, considering PMN cells and all markers used in the immunohistochemical analysis, including the CD 163+/MAC 387+ cells ratio (Tables 5 and 6, Figures 3, 4, and 5).

**Figure 6:**
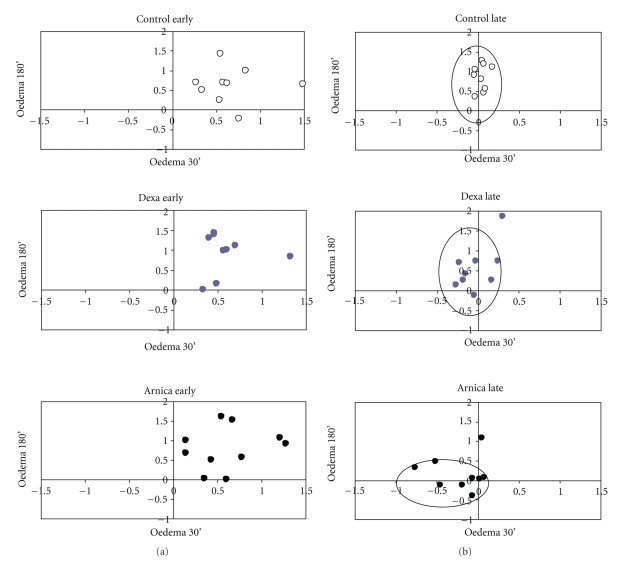
Scattering plotting showing different trends of oedema time evolution between both subgroups. The white points represent control, the black points represent *Arnica montana* 6cH, and the grey points represent dexamethasone. In each graphic, the *X*-axis shows the oedema at 30 minutes before carrageenan injection and the *Y*-axis shows the oedema at the end of experiment. LATE = late oedema subgroup; EARLY = early oedema subgroup. Note that animals that presented late peak of oedema and were treated with *Arnica montana* 6cH are displaced to the baseline in relation to the other groups.

**Figure 7:**
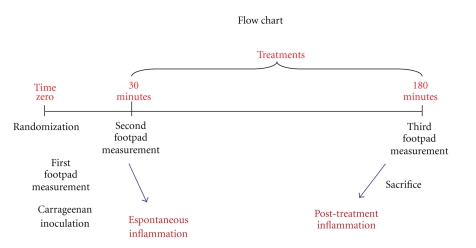
Flowchart of the experimental steps, determining two subgroups of rats according to the oedema kinetic.

Taking all data together, it is possible to illustrate the main conclusion by a scattering graph of oedema intensity, in which the different trends among groups and subgroups can be seen, after the treatment with *Arnica montana* 6cH (Figure 6). Note that animals treated with Arnica and exhibiting late oedema (*late subgroup*) are displaced to the baseline in relation to the other groups.

## 4. Discussion

Among the homeopathic medicines that have special interest in inflammation control [3, 25, 26], *Arnica montana* is one of most studied [16–20]. Nevertheless, in the recent scientific literature about homeopathy, there are many studies searching to demonstrate the efficacy of its effect, but few works are devoted to the comprehension of physiopathological changes in tissues after the exposition of animals to these medicines [6, 27, 28]. Is from this point of view that the present work was built; through a detailed study of the inflamed connective tissue, considering the subsets of cells that migrate to it, the vessels behavior, and time-dependent dynamic of this process under the action of *Arnica montana* 6cH. Interestingly, some effects observed are in accordance with previous observed results, such as the overture of lymphatic vessels and the consequent reduction of oedema [13].

The first results obtained here point toward an interesting fact: the individual analysis of each subgroup shows the importance of temporality in the effect of *Arnica montana* 6cH; animals that presented spontaneous late peak of oedema (after 30 minutes from irritant injection) were more sensible to Arnica than the animals that presented an early peak of oedema (before 30 minutes). In this case, the oedema was even less intense than the control and dexamethasone treated groups, and this fact was concomitant with the significant reduction of mast cell degranulation and increase of lymphatic vessels diameter. Both phenomena could contribute to the reduction of macroscopic oedema and differ from dexamethasone pattern, in which lymphatic vessels area reduces passively together with the oedema reduction, as shown in Figure 2. This effect corroborates the results observed previously in [13].

Carrageenan is a polysaccharide obtained from the seaweed *Chondrus crispus* that has the ability to induce local inflammation, without systemic effects [3]. It is classically known that prostaglandins are generated during the oedema formation after subcutaneous carrageenan injection by migrated neutrophils [29]. The observation of antioedema effect of *Arnica montana* 6cH exclusively in animals that presented the late pattern of acute inflammation points toward the participation of different chemical mediators in controlling pathways, as demonstrated before in [10, 11]. In this sense, histamine and prostaglandin, mediators whose peak of action occurs toward 15–30 minutes, probably modulate this process in the late oedema subgroup, instead of mediators with a very precocious action, such as bradykinin, whose peak is about 10 minutes after releasing [24, 30].

Experimental models designed specially to demonstrate the importance of individual variation in the effect of homeopathic medicine were already described before [5, 31, 32], but never in an inflammation protocol. Individuality means specificity in homeopathic medicine; in Rocha 2008 [5], only the naturally hyperactive rats were responsible to *Rhus toxicodendron* 200cH treatment, regarding their behavior in an open-field device. On the other hand, Soares 2007 [32] observed that only hypoactive rats were responsible to *Bryonia alba* 200cH. These complementary studies show results compatible to the respective *materia medica*. The systematic comparison of both studies and others is seen in [28].

Regarding to PMN and mononuclear leukocyte sub-types cell migration, no difference among groups and subgroups was seen, nor the CD163+/MAC387+ ratio revealed any change in the dynamic of macrophage maturation in the inflammatory site. The CD 163 is a transmembrane glycoprotein type I, of 175 kD, also known as ED2 protein. Its presence has been described in the mature macrophage surface, but also in a less intense manner in immature monocytes and other mononuclear cells in humans. The expression of ED 2 is associated to the iron metabolism and increases during the later phases of inflammation, because positive cells have a modulator role, releasing autocrine IL10 [33–35]. The presence of T and B lymphocytes into the inflammatory site could be related to a certain modulator action [23, 24], which has been suggested in previous studies about *Arnica montana* 6cH [13], but not confirmed herein.

The expression of adhesion molecules CD 54 (ICAM-1) was greater in animals treated with *Arnica montana* 6cH that presented early pattern of inflammation and was associated to a greater percentage of degranulated mast cells in the inflamed tissue, but not to a greater leukocyte migration. Although no mechanistic explanation could be done in this step of the study, it seems that the global results are split in two main patterns: those of regulating vascular dynamics, evident in the *late oedema subgroup*, and those of regulating cell events, evident in the *early oedema subgroup*, suggesting an individual-dependent qualitative adjustment of vascular and cellular inflammation events by *Arnica montana* 6cH, as seen in Figure 6. These different patterns of variability, oscillating according to the previous state of the tested biological system, have been experimentally described in other experimental contexts with different homeopathic preparations [26, 36–38].

In conclusion, the two proposed hypotheses could be highlighted: (a) there is no selective modulation of leukocyte subsets migration *by Arnica montana* 6cH treatment, but only vascular regulations, regarding lymphatic absorption, CD54 expression, and histamine degranulation and (b) there is a clear interference of the individual kinetic variation in vascular events after treatment with *Arnica montana* 6cH.

##  Conflict of Interests

There is no conflict of interests related to this work.

## Figures and Tables

**Table 1:** Groups and treatments.

Group	Treatment	Administration
*Arnica montana* 6cH	each 15 minutes, between 30 and 180 minutes from inoculation, orally, using automatic pipette	10 *μ*L/100 g body weight per administration (total of 8 administrations)
5% hydroalcoholic solution	idem	10 *μ*L/100 g body weight per administration (total of 8 administrations)
Dexamethasone (Azium)	idem	10 *μ*L/100 g body weight per administration (the total dose of 4 mg/kg was fractioned along 8 administrations)

**Table 2:** Schedule of groups and subgroups post-classified after the first oedema measurement (30 minutes).

Groups	Subgroups	Oedema peak
Arnica montana 6cH (*n* = 20)	Early oedema (*n* = 10)	Up to 30 minutes (pretreatment)
	Late oedema (*n* = 10)	Between 30 and 180 minutes (post-treatment)
Dexamethasone (4 mg/kg) (*n* = 20)	Early oedema (*n* = 10)	Up to 30 minutes (pretreatment)
	Late oedema (*n* = 10)	Between 30 and 180 minutes (post-treatment)
5% Hydroalcoholic solution (*n* = 20)	Early oedema (*n* = 10)	Up to 30 minutes (pretreatment)
	Late oedema (*n* = 10)	Between 30 and 180 minutes (post-treatment)

**Table 3:** Markers used in the immunohistochemistry.

Marker	Cell	Supplier	Molecular target	Clone	Origin species/target	Dilution
Anti-CD54	Activated endothelial cell	Serotec	Adhesion molecule (ICAM 1)	1A29	Mouse-rat	1 : 10 (5 *μ*g/mL)
Anti-CD18	Leukocytes	Serotec	Adhesion molecule (Integrin *β*2)	WT.3	Mouse-rat	1 : 10 (5 *μ*g/mL)
Anti-CD163	Monocytes and macrophages	Serotec	Surface glycoprotein (ED2)	ED2	Mouse-rat	1 : 10 (5 *μ*g/mL)
Anti-MAC 387	Moncytes, macrophages	AbCAM	Intracytoplasmic protein (calprotectin)	polyclonal	Rabbit-rat	1 : 20 (50 *μ*g/mL)
Anti-CD45RA	B Lymphocytes	Serotec	Surface protein (LCA)	OX-33(B cells only)	Mouse-rat	1 : 10 (5 *μ*g/mL)
Anti-CD3	T Lymphocytes	AbCAM	TCR-associated protein	polyclonal	Rabbit-rat	1 : 5 (40 *μ*g/mL)

**Table 4:** Criteria of score attribution for different patterns of endothelial cell marking.

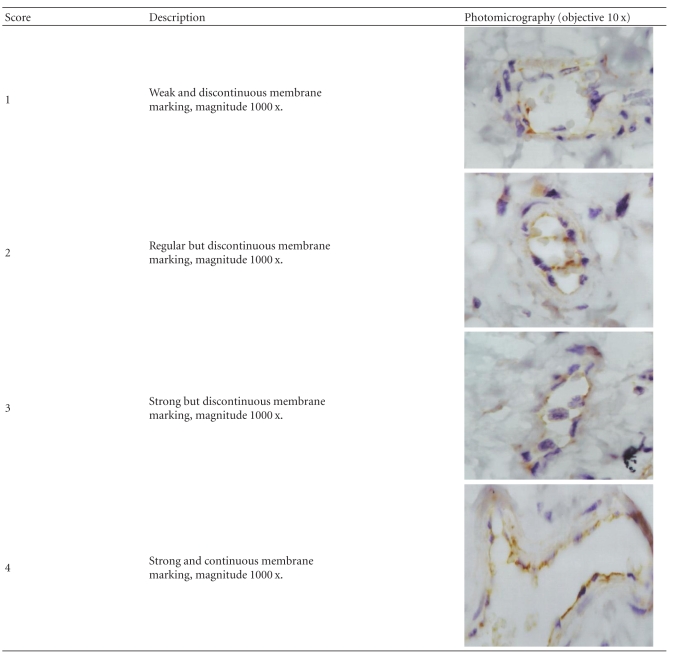

**Table 5:** General view of results obtained in rats that developed spontaneous peak of oedema between 0 and 30 minutes (*early oedema subgroup*) after the injection of 1% carrageenan into the footpad. *Kruskal-Wallis, *P* ≤ .05; ^#^ *X* ^2^, *P* = .0001 in relation to control. ^##^ANOVA, Tuckey-Krammer, *P* ≤ .05 in relation to the late oedema subgroup (Table 6).

Early oedema subgroup	Control (vehicle)	*Arnica montana* 6cH	Dexamethasone (4 mg/kg)
Paw oedema (180 min)	0.63 ± 0.45^a^	0.78 ± 0.54^##^	0.89 ± 0.48^##^
Degranulated mast cells	14%	20%^#^	16%
Lymphatic vessels diameter	101.12 ± 45.25^b^	108.08 ± 84.33	105.95 ± 63.06
CD 54	2 (2–6)^c^	5 (3–8)*	4 (3–6)
PMN	158.11 ± 29.88^d^	172.30 ± 21.60	175.90 ± 32.52
CD45RA	23.5 ± 35.34	26.33 ± 16.37	26.25 ± 13.79
CD18	62.11 ± 24.63	52.11 ± 17.87	72.88 ± 21.58
CD163	37.28 ± 6.42	33.22 ± 9.56	32.66 ± 8.12
MAC 387	46.87 ± 7.82	48.8 ± 10.11	50.9 ± 8.84
CD3	11.11 ± 4.04	13.8 ± 9.39	12.2 ± 4.13
CD163/MAC387 ratio	0.51	0.56	0.60

^a^mean ± standard deviation; millimeters.

^b^mean ± standard deviation; pixels per field.

^c^median and interval; scores attributed by two independent observers.

^d^mean ± standard deviation; cells per field.

**Table 6:** General view of results obtained in rats that developed spontaneous peak of oedema between 30 and 180 minutes (*late oedema subgroup*) after the injection of 1% carrageenan into the footpad. *Kruskal-Wallis, *P* ≤ .05; **ANOVA, *P* = .01; ^#^ *X* ^2^, *P* = .0001 in relation to control.

Late oedema subgroup	Control (vehicle)	*Arnica montana* 6cH	Dexamethasone (4 mg/kg)
Paw oedema (180 min)	0.84 ± 0.33^a^	0.07 ± 0.48**	0.51 ± 0.55
Degranulated mast cells	24%	17%^#^	16%^#^
Lymphatic vessels diameter	108.73 ± 65.34^b^	147.32 ± 113.71*	80.13 ± 44.64
CD 54	4 (2–7)^c^	4.5 (2–6)	2 (2–5)
PMN	176.33 ± 29.95^d^	185.60 ± 17.16	179.37 ± 22.67
CD45RA	22.85 ± 17.12	22.77 ± 16.20	25.88 ± 20.27
CD18	62 ± 13.68	56.8 ± 23.58	63.11 ± 18.82
CD163	31.5 ± 14.84	35.66 ± 10.34	30.11 ± 12.45
MAC 387	47.77 ± 8.24	45.2 ± 11.53	51.88 ± 13.52
CD3	12.66 ± 7.98	15.2 ± 7.50	14.88 ± 5.37
CD163/MAC387ratio	0.60	0.50	0.66

^a^mean ± standard deviation; millimeters.

^b^mean ± standard deviation; pixels per field.

^c^median and interval; scores attributed by two independent observers.

^d^mean ± standard deviation; cells per field.
